# Royal Infirmary, Edinburgh, and Insured Persons

**Published:** 1913-07-26

**Authors:** W. S. Caw

**Affiliations:** Treasurer and Clerk. Royal Infirmary, Edinburgh.


*To the Editor of* The Hospital.

### Royal Infirmary, Edinburgh, and Insured Persons.

*To the Editor of* The Hospital.

Sir,—I am directed by the managers of this institution to say that certain remarks by Councillor Macpherson at the recent meeting of the British Hospitals Association at Oxford, as reported in your issue of the 5th inst., are not in accordance with fact. The statements taken exception to are (1) that "the governors resolved to give notice that insured persons would not now be treated in the Royal Infirmary." The actual resolution adopted by the board on July 15, 1912, was as follows: "That, in present circumstances, insured persons, being entitled to medical benefits under the Insurance Act, should not be treated in the out-patient departments of the hospital except in accident cases, urgent cases, and suitable consultation cases." Insured persons requiring indoor treatment were all to be and have been looked upon as urgent cases, and they have been admitted without question. The only effect of the resolution quoted above has been to leave the trivial outdoor cases to be dealt with by the doctors provided under the Act. (2) The other statement is that "the governors met and decided to suspend any action for six months until the working of the Act became better known." No such resolution was ever come to. A motion to that effect was submitted to a meeting of the board last winter by Councillor Macpherson, but it was not considered, the board holding, by a majority, that it was not a competent motion, in view of the fact that the managers' resolution had been submitted to, approved of, and confirmed by the Court of Contributors.—I am, yours faithfully,

W. S. Caw,

Royal Infirmary, Edinburgh. Treasurer and Clerk.

July 21, 1913.

